# Stable Transmission of *Dirofilaria repens* Nematodes, Northern Germany

**DOI:** 10.3201/eid2002.131003

**Published:** 2014-02

**Authors:** Christina Czajka, Norbert Becker, Hanna Jöst, Sven Poppert, Jonas Schmidt-Chanasit, Andreas Krüger, Egbert Tannich

**Affiliations:** Kommunale Aktionsgemeinschft zur Bekämpfung der Stechmückenplage, Waldsee, Germany (C. Czajka, N. Becker, H. Jöst);; Bernhard Nocht Institute for Tropical Medicine/World Health Organization Collaborating Centre for Arbovirus and Haemorrhagic Fever Reference and Research, Hamburg, Germany (C. Czajka, H. Jöst, S. Poppert, J. Schmidt-Chanasit, E. Tannich);; German Centre for Infection Research, Hamburg (H. Jöst, J. Schmidt-Chanasit, E. Tannich);; Bundeswehr Hospital Hamburg, Hamburg (A. Krüger)

**Keywords:** transmission, Dirofilaria repens, nematodes, Germany, dirofilariosis, zoonoses, parasites

**To the Editor:** Dirofilariosis caused by infection with the filarial nematode *Dirofilaria repens* is considered an emerging zoonosis in Europe (*1*). The main reservoirs for this parasite are dogs and other carnivores. As is the case for other filarial species, mosquitoes transmit infectious third-stage larvae, which develop into fertile adults in their definitive vertebrate hosts. Humans may become infected as aberrant hosts, and, in most cases, these worms remain infertile (*1*,*2*). Infections in humans usually manifest as subcutaneous nodules, which are caused by developing worms that are trapped by immune mechanisms (*2*). Subcutaneous migration of a worm may result in local swelling. Severe clinical manifestations have also been reported and may affect various organs, including the brain, lung, and eye (*2*,*3*). Eye infections are found in particular during the migratory phase of the parasite. 

Transmission of *D. repens* occurs in various regions of the Old World, including Europe, Africa, and Asia. The primary areas in Europe to which *D. repens* is endemic are countries of the Mediterranean region, where warm temperatures enable development of infectious third-stage larvae in mosquitoes. However, during the past decade, several autochthonous cases of canine and human dirofilariasis have been reported from countries farther north, including Austria, the Czech Republic, and Poland (*4*–*6*). Several factors might be responsible for this spread to the north, including climate change and increased translocation of dogs from southern to central Europe.

Until recently, central Europe, including Germany, was not considered a region to which *D. repens* was endemic. However, a survey in 44 hunting dogs from the Upper Rhine region showed that 3 animals were positive for *D. repens* microfilariae (*7*). In addition, testing of a kennel of sled dogs located in the federal state of Brandenburg, near Berlin, found 8 of 28 animals were infected with the parasite (*8*).

To determine whether local transmission of *D. repens* is taking place in Germany, we retrospectively analyzed 74,547 mosquitoes from a large-scale German mosquito-borne virus surveillance program for the presence of filarial DNA. Mosquitoes were collected during the main animal-trapping seasons (May–September) 2011 and 2012 by using carbon dioxide–baited encephalitis virus surveillance or gravid traps (*9*). Mosquitoes were collected from 55 trapping sites located in 9 federal states in the southwest and northeastern parts of Germany (Figure), frozen at −70°C, and transported to the laboratory at the Bernhard Nocht Institute, where they were classified morphologically to the species level. Up to 25 mosquitoes of the same species from individual collection sites were subsequently combined to create a total of 4,113 pools. These pools comprised a broad range of ornithophilic and mammalophilic mosquitoes common in Germany; *Culex* spp. and *Aedes vexans* were the most abundant species. 

**Figure:**
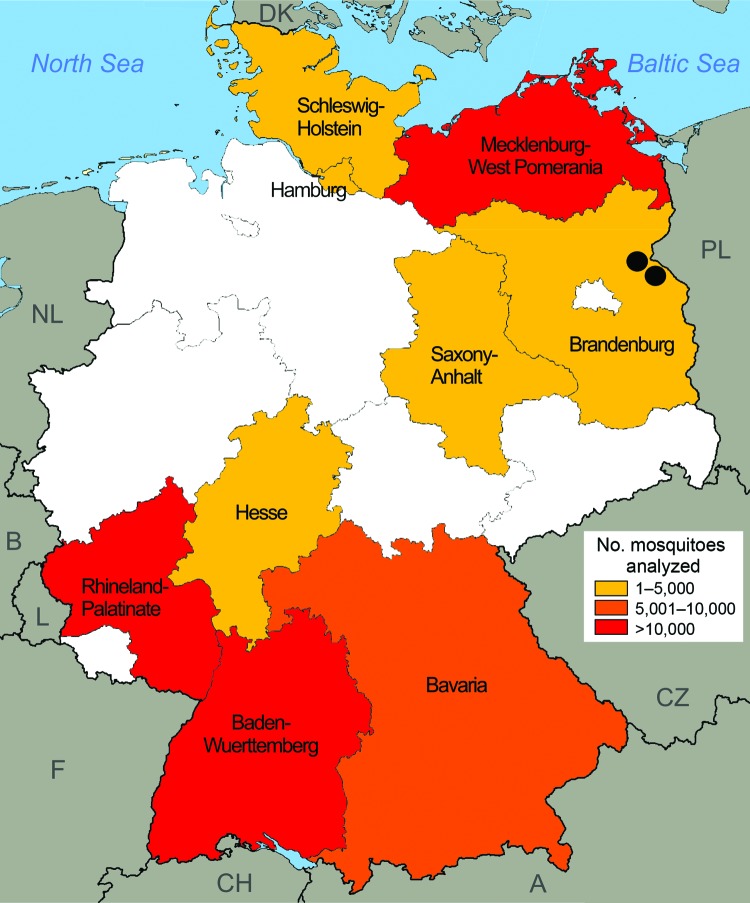
Origin of mosquito samples analyzed for infection with *Dirofilaria repens*, by federal state, Germany, May–September 2011 and 2012 (n = 74,547). Black dots indicate collection locations of mosquitoes that tested positive for *D. repens* DNA.

Nucleic acids were extracted from each mosquito pool, and the presence of filarial DNA was determined by PCR using a set of generic primers that amplify DNA from a broad range of filarial species, including those affecting birds and other animals (*9*). A total of 1,050 (25.5%) of the 4,113 pools were found to be positive for filarial DNA. To further determine if any of these pools contained DNA from *D. repens*, we analyzed all 1,050 pools by PCR using the *D. repens*–specific primers 5′-GAGATGGCGTTTCCTCGTG-3′ and 5′-GACCATCAACACTTAAAG-3′. Four pools were positive for *D. repens* DNA; all of these pools consisted of mammalophilic mosquitoes collected at 2 trapping sites in the federal state of Brandenburg, near Oder Valley, at a latitude of 52°N (global positioning satellite coordinates 52°47′N, 14°14′E, and 52°51′N, 14°7′E). One pool, consisting of *Culiseta annulata* mosquitoes, was collected in August 2011; the other 3 pools, 2 of *Anopheles maculipennis* sensu lato and 1 of *Ae. vexans* mosquitoes, were trapped in July and August 2012. The identification of *D. repens* DNA in all 4 pools was confirmed by DNA sequencing of a 538-bp fragment of the *g*ene encoding cytochrome oxidase 1 (GenBank accession no. KF410864). Sequence comparisons indicated 99.8% identity to the same gene in a *D. repens* nematode isolate from Italy (accession no. AM749234).

In conclusion, we found mosquitoes that were positive for *D. repens* DNA at a latitude of 52°N in northern Germany; these mosquitoes, from 3 species, were collected at 2 locations in 2011 and 2012. Our findings support recent projections that have suggested increases in average temperatures could affect climatic conditions during summer sufficiently to enable development of *D. repens* larvae in central Europe at latitudes of up to 56°N (*10*). In the federal state of Brandenburg in Germany, conditions for larval development were predicted to be sufficient in each of the past 12 years. These projections, together with our finding of mosquitoes carrying *D. repens* in Brandenburg in 2 successive years and the recurrent detection of infected dogs in this area (*8*), present a strong case for the existence of stable local transmission of *D. repens* in this region of Germany.

Technical Appendix PCR analyses of mosquitoes from various federal states of Germany for the presence of filarial DNA.
