# Supplementary material for: Stable Transmission of Dirofilaria repens Nematodes, Northern Germany
Source: Emerg Infect Dis. 2014 Feb;20(2):329–31. doi: 10.3201/eid2002.131003 (PMC3901495; doi:10.3201/eid2002.131003)
Supplement: Technical Appendix — PCR analyses of mosquitoes from various federal states of Germany for the presence of filarial DNA. [file 13-1003-Techapp-s1.pdf]

Article DOI: http://dx.doi.org/10.3201/eid2002.131003

Stable Transmission of *Dirofilaria repens* Nematodes, Northern Germany

Technical Appendix

Technical Appendix Table. PCR analyses of mosquitoes from various federal states of Germany for the presence of filarial DNA

Federal State	No. mosquitoes analyzed	No. pools analyzed	No. (%) pools positive for filarial DNA*	No. (%) pools positive for *Dirofilaria repens* DNA†
Baden-Wuerttemberg	19,007	1,231	215 (17.5)	0
Bavaria	6,555	358	99 (27.7)	0
Brandenburg	3,124	145	18 (12.4)	4 (2.8)
Hesse	743	45	15 (33.3)	0
Mecklenburg–West Pomerania	17,271	746	35 (4.6)	0
Rhineland-Palatinate	25,208	1,377	625 (45.4)	0
Saxony-Anhalt	1,333	71	22 (31.0)	0
Schleswig-Holstein/Hamburg	1,306	140	21 (15.0)	0
Total	74,547	4,113	1,050 (25.5)	4 (0.1)

*PCR was performed according to (*1*).
†PCR was performed by using primers 5'-GAGATGGCGTTTCCTCGTG-3′ and 5'-GACCATCAACACTTAAAG-3′.

Reference

1. Czajka C, Becker N, Poppert S, Jöst H, Schmidt-Chanasit J, Krueger A. Molecular detection of *Setaria tundra* (Nematoda: Filarioidea) and an unidentified filarial species in mosquitoes in Germany. Parasit Vectors. 2012;5:14. PubMed http://dx.doi.org/10.1186/1756-3305-5-14
